# Successful thrombolysis of a thrombosed prosthetic mitral valve using a synthetic tissue plasminogen activator: a case report

**DOI:** 10.1186/1752-1947-4-241

**Published:** 2010-08-03

**Authors:** Nael Al-Sarraf, Fahad Al-Shammari, Jamal Al-Fadhli, Emad Al-Shawaf

**Affiliations:** 1 Department of Cardiac Surgery, Chest Disease Hospital, Al-Jabriah, Kuwait; 2 Department of Cardiac Anaesthesia, Chest Disease Hospital, Al-Jabriah, Kuwait

## Abstract

**Introduction:**

Prosthetic valve thrombosis is a rare but life-threatening condition that requires careful evaluation and prompt treatment. While surgical intervention remains the gold standard, thrombolytic therapy is now emerging as a potential substitute. Various thrombolytic treatments including streptokinase, urokinase and recombinant tissue plasminogen activators have been reported with variable success rates. However, the data on the use of tenecteplase (a synthetic tissue plasminogen activator) is limited.

**Case presentation:**

A 44-year-old Middle Eastern man with a previously implanted prosthetic mitral valve presented with exertional dyspnea and orthopnea. Investigations revealed a thrombosed prosthetic mitral valve. Successful thrombolysis was achieved using tenecteplase which lead to the complete restoration of valve function with no risk to the patient.

**Conclusion:**

Prosthetic valve thrombosis is a rare but life threatening condition, the diagnosis of which requires a high index of suspicion. Tenecteplase can be used successfully in the management of such cases. It has proved to be useful with no extra risk to the patient.

## Introduction

Prosthetic valves thrombosis (PVT) is defined as any obstruction of the prosthesis by non-infective thrombotic material. The diagnosis of PVT is made by a combination of clinical data (heart failure, absence of prosthetic sounds, cardiogenic shock) and echocardiograpy. The traditional treatment for PVT is surgery with either thrombectomy or replacement of the prosthesis. In recent years, thrombolytic therapy has evolved as a substitute to surgery. Various thrombolytic treatments have been reported with variable success rates including streptokinase, urokinase and recombinant tissue plasminogen activators. However, the data on the use of tenecteplase (a synthetic tissue plasminogen activator) is limited [1].

## Case presentation

A 44-year-old Middle Eastern man was admitted for mitral valve replacement. He had history of atrial fibrillation and rheumatic heart disease affecting the mitral valve (MV) with consequent severe mitral stenosis. He was a non-smoker and did not consume alcohol. He had no family history of relevance. He weighed 60 kg and his height was 168 cm.

The patient underwent an uneventful mitral valve replacement using a mechanical 29 mm St Jude prosthesis. He was commenced on oral anticoagulant (warfarin) and a beta blocker. His pre-discharge transthoracic echocardiography (TTE) showed a well-functioning prosthesis with no paravalvular leak. Six months later, the patient presented to his local hospital with exertional dyspnea and orthopnea. He admitted that he had stopped taking his oral anticoagulant therapy 10 days prior to hospitalization: his international normalized ratio was 1.0.

**Figure 1:**
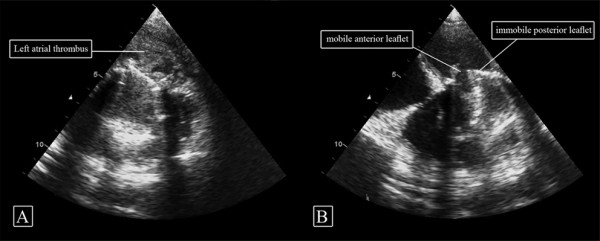
**Pre-treatment transthoracic echocardiography (TTE)**. (A) Left atrial thrombus adherent to mitral valve prosthesis and (B) TTE showing (with arrows) the two leaflets of prosthetic mitral valve (anterior leaflet mobile while posterior leaflet thrombosed and immobile).

His physical examination showed atrial fibrillation, absent prosthetic click and congested lungs but was otherwise unremarkable. Routine blood investigations were normal. TTE showed immobile posterior mitral leaflet and mobile anterior leaflet with a mean pressure gradient of 20 mmHg (peak pressure 40 mmHg) and reduced surface area of 0.7 cm^2^. He was then referred to our center. His New York Heart Association class on arrival was II and he was in a stable hemodynamic status. A repeat TTE at our center showed a huge left atrial (LA) thrombus in the posterolateral aspect of LA involving the posterior cusp of MV prosthesis which kept it in a closed position with a mobile anterior leaflet (Figure 1A and 1B). These findings were confirmed by transoesophageal echocardiography (TEE).

**Figure 2:**
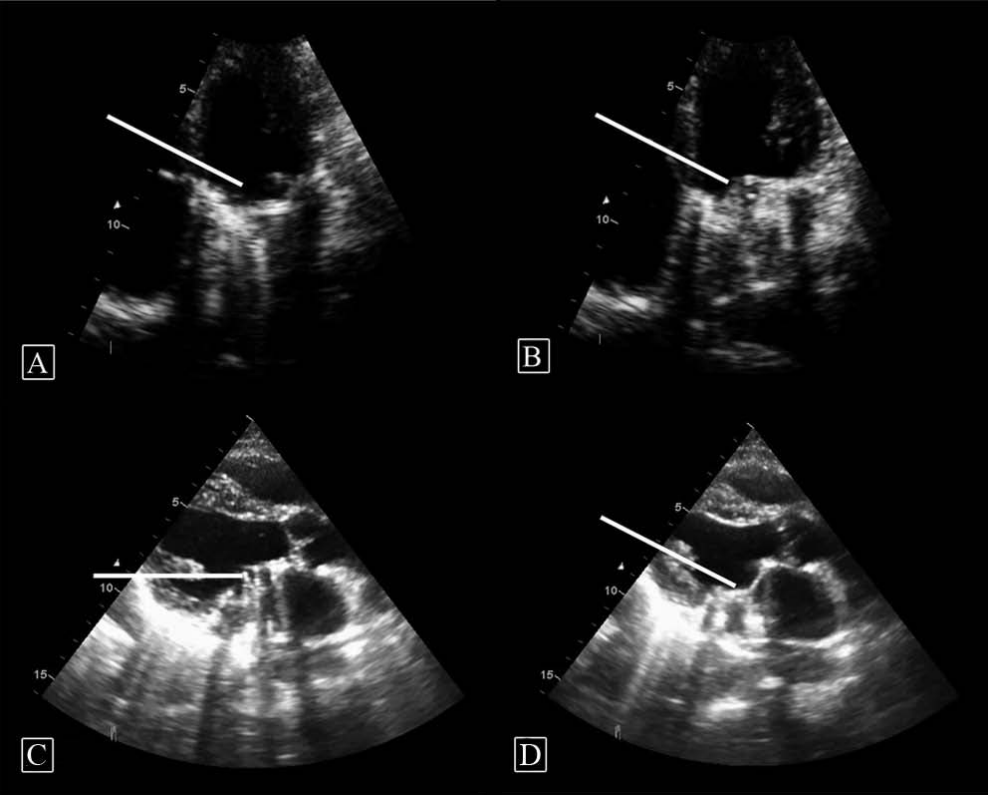
**Transthoracic echocardiography (TTE)**. Appearance at 24 hours post treatment (A and B) showing (with arrows) both mitral leaflets to be reasonably mobile: (A) in open position; (B) in closed position. (C and D) TEE appearance at 48 hours post treatment showing (with arrows) fully mobile both mitral leaflets: (C) in open position; (D) in closed position.

We proceeded with thrombolytic treatment using tenecteplase (Boehringer Ingelheim, Germany) in a slow intravenous infusion of 1.25 mg/h (dose was 0.5 mg/kg and the patient body weight was 60 kg). The patient was closely monitored in an intensive care unit for any sign of cerebral embolism or bleeding. Thrombolysis was continuous for 48 hours. Following 24 hours of tenecteplase treatment, a repeat TTE showed recovery of most of the MV mobility (Figure 2 Figure 2A and 2B) with a significant decrease in LA thrombus size and a recovering blood flow across the MV. The MV area was 2.7 cm^2 ^with no paravalvular leak and pericardial effusion.

After 48 hours of treatment, a TEE showed well functioning MV prosthesis with no residual thrombus (Figure 2C and 2D). The surface area was 2.8 cm^2 ^and the peak pressure gradient across MV was 6 mmHg with a mean pressure gradient of 2 mmHg. Following thrombolysis, intravenous anticoagulation (unfractionated heparin) was started as a continuous infusion together with oral anticoagulation therapy (warfarin). The combination was then continued until his International Normalised Rate was in the therapeutic range and then heparin was discontinued. The patient was discharged home in a stable condition with no complications. He remained well four months later.

## Discussion

PVT is defined as any obstruction of prosthesis by non-infective thrombotic material. It has an estimated incidence of 0.03% - 4.3% per year [2] and is reported to occur in 0.5% - 8% of the left-sided prosthetic valves and in up to 20% of tricuspid prostheses [3,4]. The most common cause of PVT is inadequate anticoagulant therapy (in up to 82% of cases) [3]. Other pathogenic factors include: mitral position of the prosthesis; type of prosthesis; atrial fibrillation; left atrial enlargement; ventricular dysfunction; and multiple valve replacements [5]. Diagnosis of prosthetic heart valve thrombosis is made by a combination of clinical data (heart failure, absence of prosthetic sounds, cardiogenic shock), fluoroscopic examination (abnormal mobility of tilting disks) and echocardiographic (both transthoracic and transoesophageal ) abnormalities (high prosthetic gradient, reduction of effective valve orifice area, lack of disk mobility and detection of thrombotic mass adherent to the prosthesis ) [3,6].

Management of PVT remains controversial. There are currently no randomized controlled trials favoring surgery over thrombolysis and vice versa. Surgery, in the form of thrombectomy or valve replacement, remains the treatment of choice but carries a significant mortality ranging from 4.7% to 20% [2,3,7]. Thrombolysis, on the other hand, has emerged as an attractive alternative with reported success rates in the region of 75%-88% for PVT [2]. Our patient had stable hemodynamics which gave us a window of opportunity to use thrombolysis therapy. If this had been unsuccessful we would have proceeded with surgery. In addition, the patient had received his first MV replacement six months previously and we felt that, by giving him a trial of thrombolysis, we would have spared him the added burden of undergoing surgery twice in a short time period. The current treatment algorithms suggested by some authors include using thrombolysis for right sided PVT while using surgery for left-sided PVT [8,9]. They also recommend using thrombolytic therapy in left-sided PVT if surgery is contraindicated or if the patient is critically ill [9]. Others have reported that patients in the New York Heart Association functional classes I and II achieve the best results with thrombolytic therapy with the lowest incidence of peripheral embolism [10]. In our case, the use of tenecteplase proved useful in a stable patient with no increased risk. Following thrombolysis, he was placed on oral anticoagulant only. We have not started him on low-dose aspirin because there was no evidence of concomitant coronary artery disease and his thrombosed valve was caused solely by his discontinuation of warfarin. However, recent guidelines have suggested that in such cases the use of low-dose aspirin, in addition to oral anticoagulant, may decrease the chance of recurrence [11].

Thrombolytics reported in the literature are streptokinase, urokinase and recombinant t-PA (alteplase) [5]. There are no studies comparing these different thrombolytic agents in PVT [3,7]. The most important complications of thrombolytic therapy are thromboembolic events and cerebral hemorrhage. Thromboembolism is more frequent in left-sided prosthesis with an incidence of 9%-20%. Embolic complications occur in two forms: peripheral embolism and cerebral. Cerebral embolism has an overall incidence 3%-10% (more in the presence of atrial fibrillation). Cerebral hemorrhage rates fluctuate between 0%-3% [3,7]. Tenecteplase is a synthetically engineered variant of alteplase designed to have increased fibrin specificity, greater efficacy, increased resistance to plasminogen activator inhibitor-1 (PAI-1) and a longer half-life. It has been used extensively in acute myocardial infarction (including in our institute) but its use in PVT treatment has rarely been reported [1,3]. One advantage of tenecteplase is that its dosing is based on actual body weight which enhances both safety and efficacy outcomes by avoiding wider fluctuations in the drug plasma concentration. Compared to recombinant t-PA (for example, alteplase), tenecteplase use leads to lower rates of bleeding complications and a decreased risk of cerebral hemorrhage among high risk patients [12]. Tenecteplase, contrary to recombinant tissue plasminogen activators, has been synthetically modified which in turn has important clinical applications. The increased fibrin specificity theoretically enhances the enzymatic activity at the clot and reduces systemic fibrinolysis. Furthermore, the increased resistance to PAI-1, an enzyme secreted by platelets that inhibit thrombolytics, may enhance the efficacy of tenecteplase. This drug proved useful in our case where successful thrombolysis was achieved for mitral PVT with no increased risk to the patient. We elected to use it in a slow infusion rate rather than a bolus in order to potentially reduce the risk of breaking up the thrombus into large emboli and to potentially reduce the risk of cerebral bleed.

## Conclusion

We present a rare case of mitral PVT which was successfully treated with tenecteplase. Its use proved to be useful with no extra risk to the patient.

## Abbreviations

LA: left atrial; MV: mitral valve; PAI-1: plasminogen activator inhibitor-1; PVT: prosthetic valve thrombosis; TEE: trans-esophageal echocardiography; t-PA: tissue plasminogen activator; TTE: trans-thoracic echocardiography.

## Consent

Written informed consent was obtained from the patient for publication of this case report and any accompanying images. A copy of the written consent is available for review by the Editor-in-Chief of this journal.

## Competing interests

The authors declare that they have no competing interests.

## Authors' contributions

NAS wrote the initial manuscript. FAS and JAF were major contributors in the writing of the manuscript. NAS and EAS conducted the literature review in this paper and edited the paper. All authors read and approved the final manuscript

## References

[B1] Ferreiro-Gutiérrez JL Ariza-Solé A Mañas-Jiménez P Ruiz-Majoral A Repeated thrombolysis with tenecteplase as a bridge to valvular replacement in a case of preoclusive mitral prosthetic thrombosis Med Clin (Barc) 2009 133 10 402 403 10.1016/j.medcli.2008.06.019 19747593

[B2] Bollag L Attenhofer Jost CH Vogt PR Linka AZ Rickli H Oechslin E Pretre R Dubach P Turina F Jenni R Symptomatic mechanical heart valve thrombosis: high morbidity and mortality despite successful treatment options Swiss Med Wkly 2001 131 109 116 11416965 10.4414/smw.2001.09685

[B3] Caceres-Loriga FM Perez-Lopez H Santos-Gracia J Morlans-Hernandez K Prosthetic heart valve thrombosis: pathogenesis, diagnosis and management Int J Cardiol 2006 110 1 6 10.1016/j.ijcard.2005.06.051 16038994

[B4] Ozkan M Kaymaz C Kirma C Sönmez K Ozdemir N Balkanay M Yakut C Deligonul U Intravenous thrombolytic treatment of mechanical prosthetic valve thrombosis: A study using serial transoesophageal echocardiography J Am Coll Cardiol 2000 35 1881 1889 10.1016/S0735-1097(00)00654-9 10841239

[B5] Caceres-Loriga FM Perez-Lopez H Morlans-Hernandez K Facundo-Sanchez H Santos-Gracia J Valiente-Mustelier J Rodiles-Aldana F Marrero-Mirayaga MA Betancourt BY Lopez-Saura P Thrombolysis as a first choice therapy in prosthetic heart valve thrombosis. A study of 68 patients J Thromb Thrombolysis 2006 21 185 190 10.1007/s11239-006-4969-y 16622616

[B6] Montorsi P Cavoretto D Alimento M Muratori M Pepi M Prosthetic mitral valve thrombosis: can fluoroscopy predict the efficacy of thrombolytic treatment? Circulation 2003 108 suppl 2 79 84 12970213 10.1161/01.cir.0000087900.45365.45

[B7] Das M Twomey D Al Khaddour A Dunning J Is thrombolysis or surgery the best option for acute prosthetic valve thrombosis? Interact Cardiovasc Thorac Surg 2007 6 806 812 10.1510/icvts.2007.165399 17846078

[B8] Roudaut R Lafitte S Roudaut MR Courtault C Perron JM Jais C Pillois X Coste P DeMaria A Fibrinolysis of mechanical prosthetic valve thrombosis: a single-center study of 127 cases J Am Coll Cardiol 2003 41 4 653 658 10.1016/S0735-1097(02)02872-3 12598078

[B9] Lengyel M Fuster V Keltai M Roudaut R Schulte HD Seward JB Chesebro JH Turpie AG Guidelines for the management of left-sided prosthetic valve thrombosis: a role for thrombolytic therapy. Consensus conference on prosthetic valve thrombosis J Am Coll Cardiol 1997 30 6 1521 1526 10.1016/S0735-1097(97)00345-8 9362411

[B10] Manteiga R Carlos Souto J Altes A Mateo J Aris A Dominguez JM Borras X Carreras F Fontcuberta J Short-course thrombolysis as the first line therapy for cardiac valve thrombosis J Thorac Cardiovasc Surg 1998 115 4 780 784 10.1016/S0022-5223(98)70355-1 9576210

[B11] Bonow RO Carabello BA Chatterjee K de Leon AC Jr Faxon DP Freed MD Gaasch WH Lytle BW Nishimura RA O'Gara PT O'Rourke RA Otto CM Shah PM Shanewise JS 2006 Writing Committee Members, American College of Cardiology/American Heart Association Task Force 2008 Focused update incorporated into the ACC/AHA 200 guidelines for the management of patients with valvular heart disease: a report of the American College of Cardiology/American Heart Association Task Force on Practice Guidelines (Writing Committee to Revise the 1998 Guidelines for the Management of Patients With Valvular Heart Disease): endorsed by the Society of Cardiovascular Anesthesiologists, Society for Cardiovascular Angiography and Interventions, and Society of Thoracic Surgeons Circulation 2008 118 15 e523 661 10.1161/CIRCULATIONAHA.108.190748 18820172

[B12] Guerra DR Karha J Gibson CM Safety and efficacy of tenecteplase in acute myocardial infarction Expert Opin Pharmacother 2003 4 5 791 798 10.1517/14656566.4.5.791 12740001

